# Fluoride as a Potential Repressor of Glycogen Metabolism in Skeletal Muscle Cell Line CCL136

**DOI:** 10.3390/molecules28166065

**Published:** 2023-08-15

**Authors:** Izabela Gutowska, Agnieszka Maruszewska, Marta Skórka-Majewicz, Agnieszka Kempińska-Podhorodecka, Agnieszka Kolasa, Agata Wszołek, Irena Baranowska-Bosiacka, Wojciech Żwierełło

**Affiliations:** 1 Department of Medical Chemistry, Pomeranian Medical University in Szczecin, 70-111 Szczecin, Poland; marta.skorka.majewicz@pum.edu.pl (M.S.-M.); wojciech.zwierello@pum.edu.pl (W.Ż.); 2 Department of Physiology and Biochemistry, Institute of Biology, University of Szczecin, 70-453 Szczecin, Poland; agnieszka.maruszewska@usz.edu.pl (A.M.); agata.wszolek@usz.edu.pl (A.W.); 3 Department of Medical Biology, Pomeranian Medical University in Szczecin, 70-111 Szczecin, Poland; agnieszka.kempinska.podhorodecka@pum.edu.pl; 4 Department of Histology and Embriology, Pomeranian Medical University in Szczecin, 70-111 Szczecin, Poland; agnieszka.kolasa@pum.edu.pl; 5 Department of Biochemistry, Pomeranian Medical University in Szczecin, 70-111 Szczecin, Poland; irena.baranowska.bosiacka@pum.edu.pl

**Keywords:** fluoride (F-) toxicity, glycogen phosphorylase muscle isoform (PYGM), glycogen synthase (GYS), glycogen synthase kinase (GSK), muscle glycogen metabolism

## Abstract

The exposure of humans to fluorine is connected with its presence in the air, food and water. It is well known that fluorides even at a low concentration but with long time exposure accumulate in the body and lead to numerous metabolic disorders. Fluoride is recognised as a factor modulating the energy metabolism of cells. This interaction is of particular importance in muscle cells, which are cells with high metabolic activity related to the metabolism of glucose and glycogen. In someone suffering from chronic fluoride poisoning, frequent symptoms are chronic fatigue not relieved by extra sleep or rest, muscular weakness, muscle spasms, involuntary twitching. The aim of this study was to examine the effect of fluorine at concentrations determined in blood of people environmentally exposed to fluorides on activity and expression of enzymes taking part in metabolism of muscle glycogen. CCL136 cells were cultured under standard conditions with the addition of NaF. The amount of ATP produced by the cells was determined using the HPLC method, the amount and expression of genes responsible for glycogen metabolism using WB and RT PCR methods and the amount of glycogen in cells using the fluorimetric and PAS methods. It has been shown that in CCL136 cells exposed to 1, 3 and 10 μM NaF there is a change in the energy state and expression pattern of enzymes involved in the synthesis and breakdown of glycogen. It was observed that NaF caused a decrease in ATP content in CCL136 cells. Fluoride exposure also increased glycogen deposition. These changes were accompanied by a decrease in gene expression and the level of enzymatic proteins related to glycogen metabolism: glycogen synthase, glycogen synthase kinase and glycogen phosphorylase. The results obtained shed new light on the molecular mechanisms by which fluoride acts as an environmental toxin.

## 1. Introduction

Glucose is stored inside muscle fibres as glycogen particles, which are not homogeneously distributed throughout muscle fibres, but are mainly found in three topographical regions: subsarcolemmal, intermyofibrillar and intramyofibrillar [1,2]. A large number of proteins have been reported to associate directly or indirectly with glycogen particles, including glycogen synthase (GYS), glycogen phosphorylase (PYGM) and glycogen synthase kinase 3 (GSK-3α/ß) [3,4,5].

Glycogen is a polysaccharide that is the principal storage form of glucose in animal and human cells. Glycogen is made primarily by the liver and muscles, but it can also be made by glycogenesis in most cells [6]. Glucose uptake into muscle and its subsequent storage as glycogen is a crucial factor in energy homeostasis in skeletal muscle. This process is stimulated acutely by insulin. A signaling pathway involving protein kinase B and GSK-3α/ß seems certain to have a key role in stimulating glycogen synthesis. Although glycogen synthesis is one of the classical insulin-regulated pathways, it is also regulated in an insulin-independent manner; for example, glycogen synthesis in muscle is stimulated significantly after strenuous exercise, with much of this stimulation being independent of the involvement of insulin [5]. Interestingly, glycogen also has other functions such as being an energy store, and the size of the glycogen stores affects, e.g., expression of various genes, the carbohydrate utilization, insulin sensitivity and basic cell function [7,8,9].

The exposure of humans to fluorine is connected with its presence in the air, food and water [10], and in many parts of the world where drinking water contains more than 1–1.5 ppm of fluoride, its high levels lead to fluorosis, a serious health problem [11].

It is well known that fluorides even at a low concentration but with long time exposure accumulate in the body and lead to numerous metabolic disorders [12]. Many authors described the influence of fluorides on the amount and activity of various enzymes taking part in different metabolic ducts [13,14,15]. But there are only few articles about the influence of fluorides on glycogen metabolism in the muscle, which is a very important source of glucose and then energy for muscle work [16,17,18]. Waldbott et al. [19] studied about 500 people affected by chronic fluoride toxicity and made a list of the clinical features. They noted, however, that the symptoms could have other origins, even in someone suffering from chronic fluoride poisoning: chronic fatigue not relieved by extra sleep or rest, muscular weakness, muscle spasms, involuntary twitching. Their observations were in line with the Susheela study [20].

Therefore, we decided to examine the effect of fluorides at concentrations determined in the blood of people environmentally exposed to fluorine on the activity and expression of enzymes taking part in the metabolism of muscle glycogen.

## 2. Results

### 2.1. NaF Decreased ATP Level in CCL136 Muscle Cell Line

**Figure 1:**
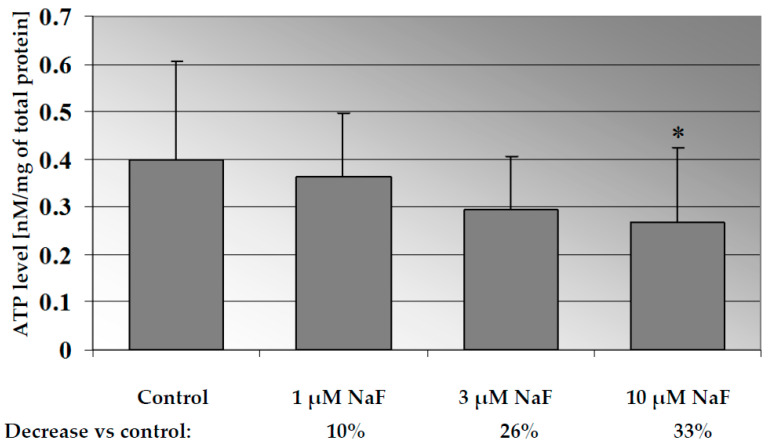
NaF decreased ATP level in muscle cells CCL136 line. Cells were cultured in high-glucose medium with 1, 3 and 10 μM NaF for 48 h and then further incubated for 30 min. with 0.1 μM insulin. After incubation, cells were harvested by scraping and ATP level was determined by HPLC assay and calculated per mg of protein. The concentration values represent mean ± SD of at least three independent experiments. The significance level of the differences observed between value found for cells treated with 10 μM NaF compared with value found for control cells: * *p* < 0.05.

Incubation of CCL136 cells with increasing concentrations of fluorides caused a significant decrease in the cellular ATP level in the dose-dependent manner (*p* = 0.028 for 10 µM NaF) (Figure 1). NaF at the concentration of 1 μM decreased the ATP level by 10% and NaF at 3- and 10 µM decreased the ATP concentration by more than 25% compared to control cells.

### 2.2. NaF Induced Glycogen Mobilisation in CCL136 Muscle Cell Line

**Figure 2:**
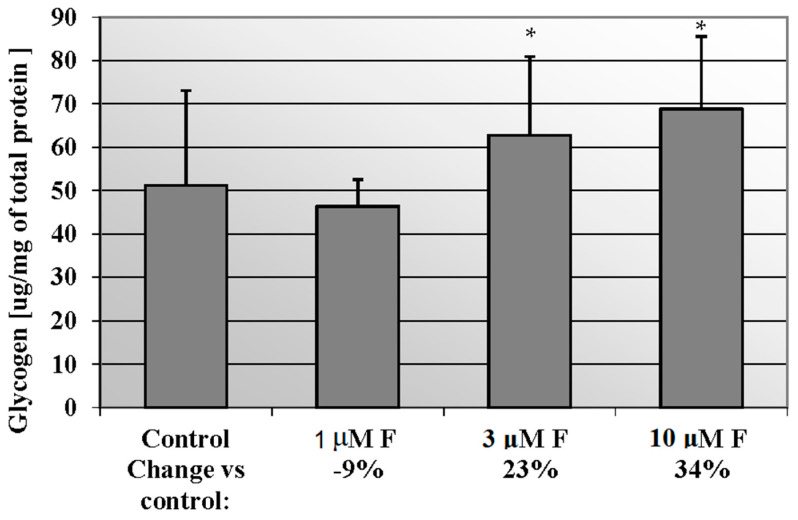
NaF induced glycogen mobilisation in muscle cells CCL136 line. Cells were cultured in high-glucose medium with 1, 3 and 10 μM NaF for 48 h and then further incubated for 30 min. with 0.1 μM insulin. After incubation, cells were harvested by scraping and glycogen concentration was determined by fluorimetric assay and calculated per milligram of protein. The concentration values represent mean ± SD of at least three independent experiments. The significance level of the differences observed between value found for cells treated with 3- and 10 μM NaF compared with value found for control cells: * *p* < 0.05.

We noted an increased concentration of glycogen in cells incubated with rising NaF concentration in culture medium (Figure 2). Statistical significance differences (*p* = 0.05) were noted between control and 3 μM NaF and 10 μM NaF. In miocytes exposed for 3 μM and 10 μM NaF, the glycogen amount was increased against control cells by 23% and 34%, respectively.

**Figure 3:**
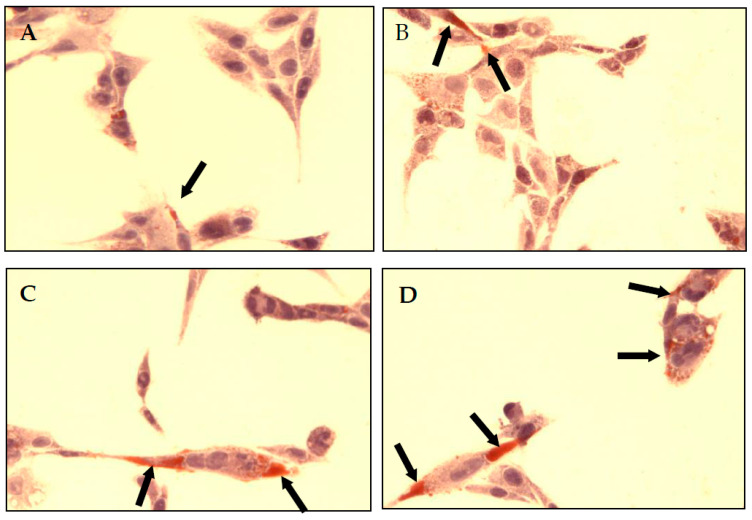
NaF increased glycogen accumulation in muscle cells CCL136 line. Imaging of glycogen localization in muscle cells CCL136 cultured without ((**A**)—the control), and with 1 μM NaF (**B**), 3 μM NaF (**C**) and 10 μM NaF (**D**). The glycogen granulation is visualised as brown stained area marked on microscopic images with black arrows. Cells were cultured in high-glucose medium with 1, 3 and 10 μM NaF for 48 h on microscope slides and then further incubated for 30 min with 0.1 μM insulin. After incubation, slides were collected and glycogen granules in cells were detected with PAS method staining as described in Materials and Methods. The preparations were examined under a fluorescence microscope.

Quantitative results were proved by the immunohistochemical method (Figure 3). PAS staining showed an increased number of glycogen granulations in CCL136 cells. Observed changes were directly proportional to NaF concentration in culture medium.

### 2.3. NaF Induce Depletion in Gene Expression of GSK-3α/β as Well as Level of Active GSK-3α/β and Its Phosphorylated Form (pGSK-3α) in CCL136 Muscle Cell Line

**Figure 4:**
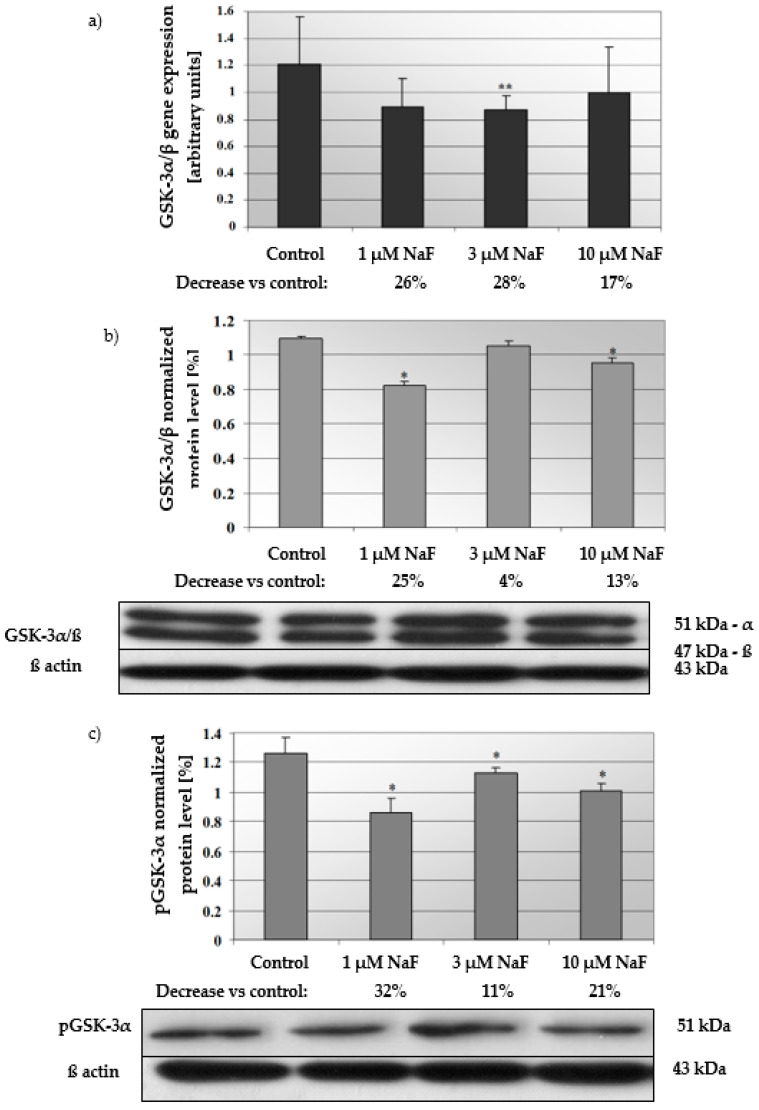
NaF caused disorders in glycogen synthase kinase 3 (GSK-3α/ß) expression, content and activation. mRNA levels (**a**), and representative Western blots and densitometric analysis of protein level (normalized to β-actin) of GSK-3α/ß (**b**), and pGSK-3α (phosphorylated, inactive form) (**c**) enzyme in CCL136 muscle cell line cultured with NaF. Cells were cultured in high-glucose medium with 1, 3 and 10 μM NaF for 48 h and then further incubated for 30 min with 0.1 μM insulin. After incubation, cells were harvested by scraping and mRNA was measured by using RealTime PCR method (*n* = 4) and protein expression by using Western Blotting method (*n* = 3). *—statistical significance vs. control (*p* < 0.03); **—in border of statistical significance vs. control (*p* < 0.07).

In cells exposed for all used NaF concentrations, mRNA and protein levels of the GSK-3α/ß enzyme were decreased (Figure 4a,b). GSK-3α/ß gene expression in cells exposed to 1, 3 and 10 μM NaF was less than in control cells by 26, 28 and 17%, respectively. Statistical significance was noted for 3 μM NaF. GSK-3α/ß protein content was decreased against control by 25, 4 and 13% in cells incubated with 1, 3 and 10 μM NaF, respectively. Statistical significance in this case was noted for 1 and 10 μM NaF.

**Figure 5:**
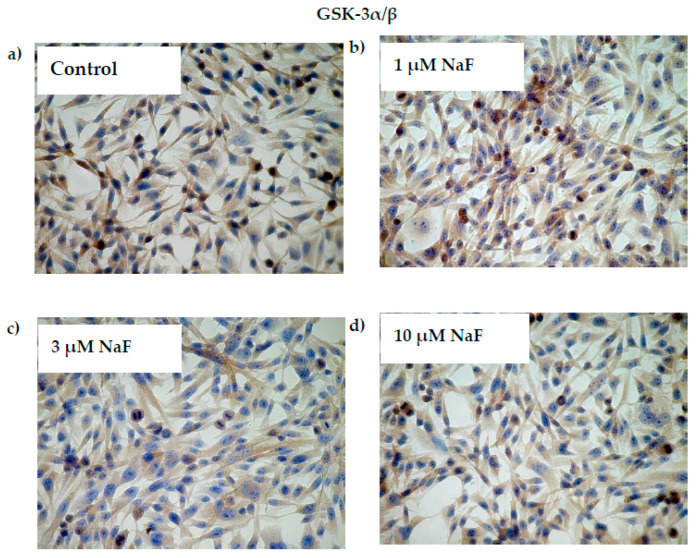

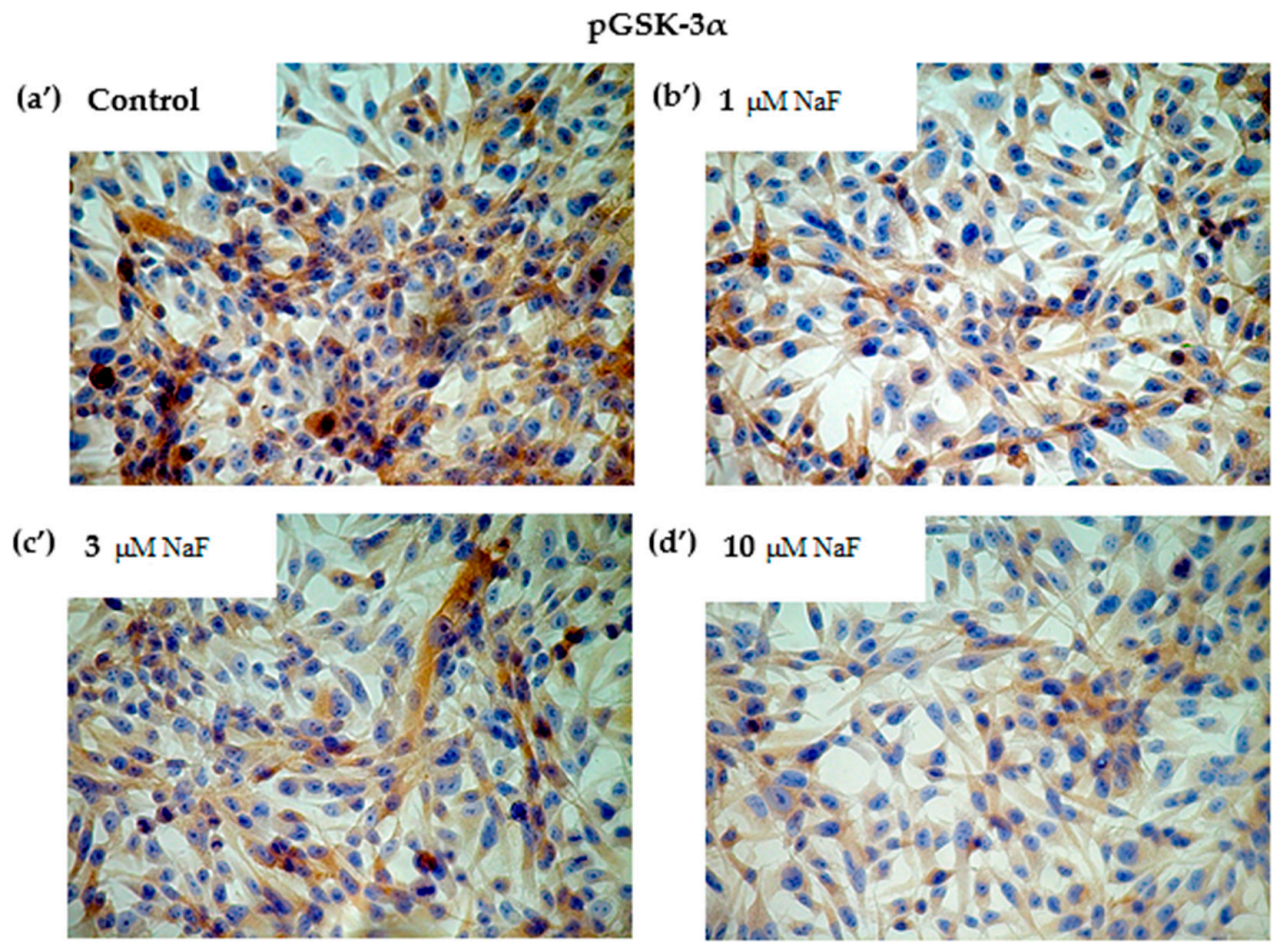
NaF decreased glycogen synthase kinase (GSK-3α/ß) enzyme and its phosphorylated form (pGSK-3α) content in muscle cells CCL136 line. Imaging (brown pigmentation) of GSK-3α/β (**a**–**d**), and pGSK-3α (**a’**–**d’**) protein depletion in cytoplasm of muscle cells CCL136 line cultured without (**a**,**a’**—the control), and with 1 μM NaF (**b**,**b’**), 3 μM NaF (**c**,**c’**) and 10 μM NaF (**d**,**d’**). Cells were cultured in high-glucose medium with 1, 3 and 10 μM NaF for 48 h on microscope slides and then further incubated for 30 min with 0.1 μM insulin. After incubation, slides were collected and cells were stained by immunocytochemistry as described in Section 4. Positive staining was determined by visual identification under microscope.

Additionally, the increased NaF concentration added to the cells caused a decrease in the protein level of the phosphorylated GSK-3α enzyme inactive form (Figure 4c). In this case, the significant decrease was noted for all used NaF concentrations in comparison to control. Namely, exposure to 1, 3 and 10 μM NaF resulted in a statistically significant reduction in pGSK-3α levels in cells by 32, 11 and 21%, respectively, compared to control cells. The obtained results were confirmed in immunohistochemical staining (Figure 5). Microscopic analysis of cells exposed to 1, 3 and 10 μM NaF revealed significant depletion in the pGSK-3α form compared to control cells.

### 2.4. NaF Decreased Gene Expression and Protein Level of GYS-1 Enzyme

**Figure 6:**
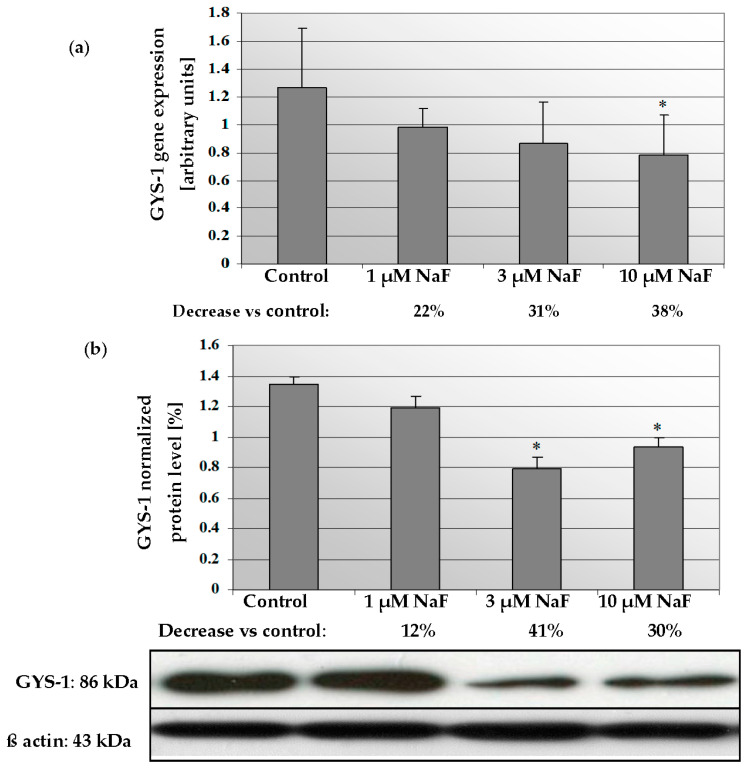
NaF caused disorders in glycogen synthase (GYS-1) expression and content. mRNA levels (**a**), and representative Western blots and densitometric analysis of protein (normalized to β-actin) of GYS-1 enzyme (**b**) in CCL136 muscle cell line cultured with NaF. Cells were cultured in high-glucose medium with 1, 3 and 10 μM NaF for 48 h and then further incubated for 30 min with 0.1 μM insulin. After incubation, cells were harvested by scraping and mRNA was measured by using RealTime PCR method (*n* = 4) and protein expression by using Western Blotting method (*n* = 3). *—statistical significance vs. control (*p* < 0.03).

In muscle CCL136 cells cultured with NaF, mRNA for GYS-1 enzyme decreased in a dose-dependent manner (Figure 6a). Exposure to 1, 3 and 10 μM NaF caused depletion of gene expression in analysed cells by 22, 31 and 38%, respectively, compared to control cells. Statistical significance was noted for 10 μM NaF, but for 1 μM, NaF decreasing was in a board of statistical significance (*p* = 0.09). The GYS-1 protein level in NaF-exposed cells was also decreased by 12, 41 and 30% against control cells with statistically significant differences for 3 and 10 μM NaF (Figure 6b).

**Figure 7:**
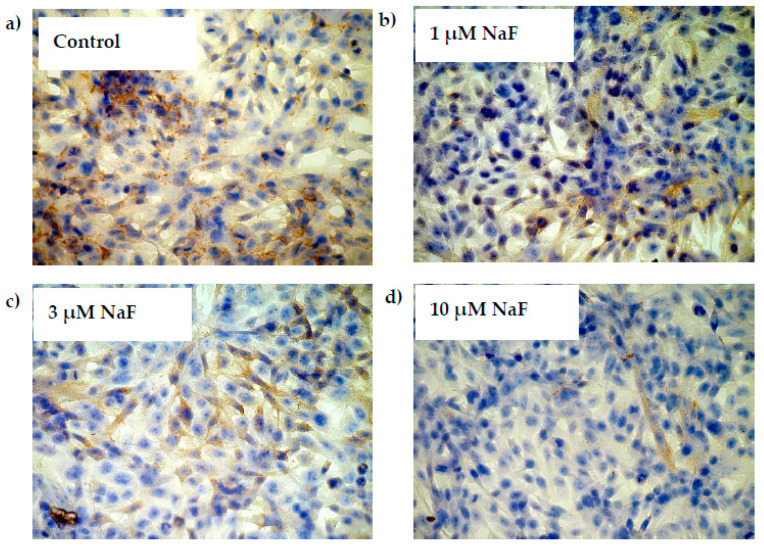
NaF decreased glycogen synthase (GYS-1) enzyme content in muscle cells CCL136 line. Imaging (brown pigmentation) of GYS-1 depletion in cytoplasm of muscle cells CCL136 cultured without ((**a**)—control), and with 1 μM NaF (**b**), 3 μM NaF (**c**) and 10 μM NaF (**d**). Cells were cultured in high-glucose medium with 1, 3 and 10 μM NaF for 48 h on microscope slides and then further incubated for 30 min. with 0.1 μM insulin. After incubation, slides were collected and cells were stained by immunocytochemistry as described in Section 4. Positive staining was determined by visual identification under microscope.

These results were confirmed by immunohistochemistry staining that revealed GYS-1 enzyme depletion in analysed cells in a dose-dependent manner (Figure 7).

### 2.5. NaF Decreased Gene Expression and Protein Level of PYGM Enzyme

**Figure 8:**
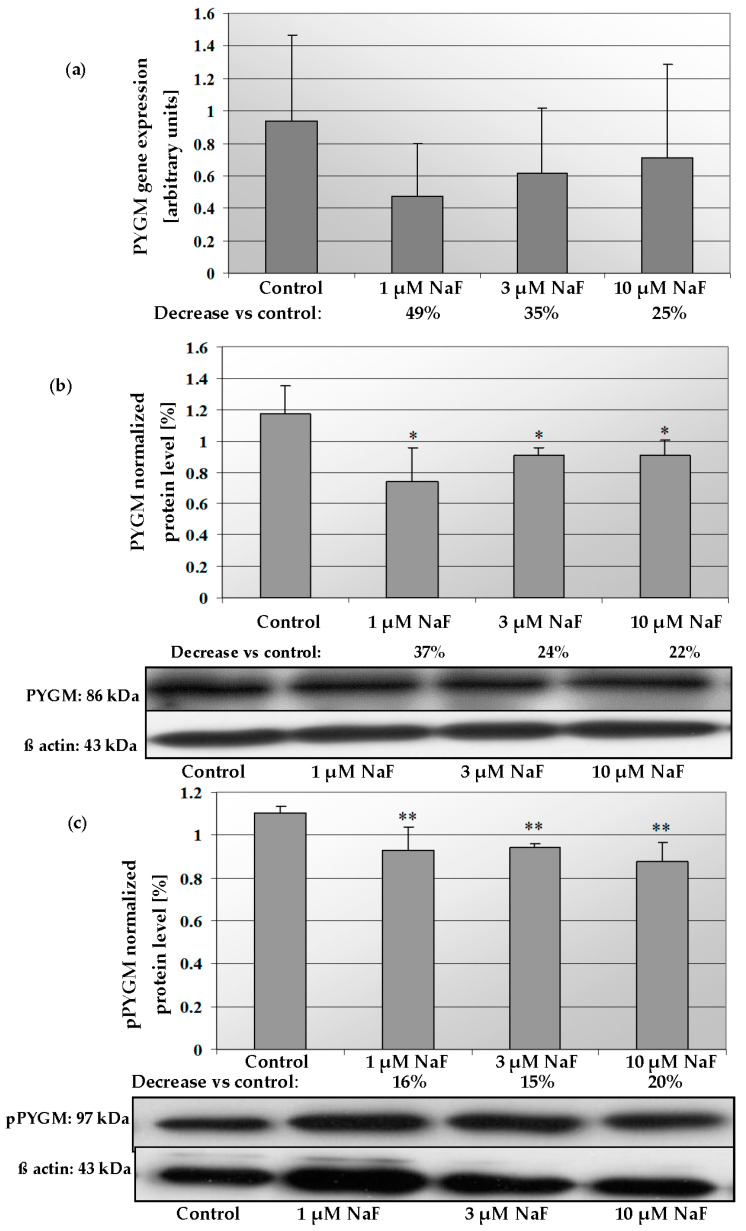
NaF caused disorders in glycogen phosphorylase (PYGM) expression, content and activation. mRNA levels (**a**), and representative Western blots and densitometric analysis of protein level (normalized to β-actin) of PYGM (**b**), and pPYGM (phosphorylated, active form) (**c**) enzyme in CCL136 muscle cell line cultured with NaF. Cells were cultured in high-glucose medium with 1, 3 and 10 μM NaF for 48 h and then further incubated for 30 min. with 0.1 μM insulin. After incubation, cells were harvested by scraping and mRNA was measured by using RealTime PCR method (*n* = 4) and protein expression by using Western Blotting method (*n* = 3). *—statistical significance vs. control (*p* < 0.03); **—in border of statistical significance vs. control (*p* < 0.07).

**Figure 9:**
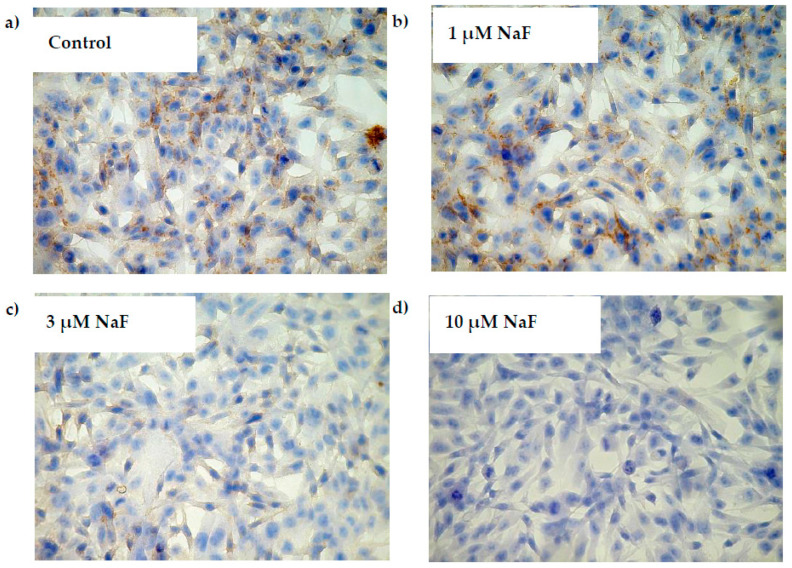
NaF decreased phosphorylated form of glycogen phosphorylase (pPYGM) enzyme content in muscle cells CCL136 line. Imaging (brown pigmentation) of pPYGM depletion in cytoplasm of muscle cells CCL136 cultured without ((**a**)—control), and with 1 μM NaF (**b**), 3 μM NaF (**c**) and 10 μM NaF (**d**). Cells were cultured in high-glucose medium with 1, 3 and 10 μM NaF for 48 h on microscope slides and then further incubated for 30 min with 0.1 μM insulin. After incubation, slides were collected and cells were stained by immunocytochemistry as described in Section 4. Positive staining was determined by visual identification under microscope.

The addition of 1, 3 and 10 μM NaF to the CCL136 muscle cell line culture influenced PYGM gene expression and enzyme level (Figure 8a,b). NaF was found to decrease the mRNA level for PYGM in cells exposed to all used fluoride concentrations. Similar activity was observed in this enzyme’s protein content. NaF at 1, 3 and 10 μM significantly decreased the intracellular PYGM level by 37, 24 and 22%, respectively. Additionally, the phosphorylated form of PYGM (pPYGM) was analysed (active form). It was shown that fluoride decreased the pPYGM protein level in a statistically significant manner (Figure 8c). The cells exposed for 1, 3 and 10 μM NaF had by 16, 15 and 20%, respectively, lower pPYGM levels compared to control cells. Molecular analyses were confirmed by immunohistochemical staining. It was shown that the intracellular pPYGM content in cells exposed for all NaF concentrations used was decreased in dose-dependent manner. NaF-treated cells staining was much lower compared to the control sample (Figure 9).

## 3. Discussion

The presence of environmental fluoride and its impact on human health is well documented (for reviews see Refs. [21,22]). At excessive exposure levels, ingestion of fluoride causes dental fluorosis, skeletal fluorosis and manifestations such as gastrointestinal, neurological and urinary problems [23]. But some authors pay attention to the disturbing symptoms of the muscular system in the case of fluorides exposure [24,25,26,27].

Fluoride is toxic to cells in a pleiotropic manner, e.g., by ROS generation, DNA damaging, inhibiting protein synthesis, disturbing mitochondrial function or cell signalling dysregulation [28,29]. One of most severe cellular activities of fluoride is ATP level depleting. Since the ATP concentration in living cells must remain within a narrow range for normal cell function, it is very important to keep the balance between ATP utilization and ATP production, and a significant depletion of ATP level impairs cell functional integrity. This should be particularly important for skeletal muscle cells that have a high ATP turnover and where energy turnover can increase dramatically during exercise [30]. Fluoride is a well-known inhibitor of glycolysis-related enzymes, as well as mitochondrial transmembrane potential disturbing factor. Moreover, fluoride is also a prooxidant agent. These activities may lead to a decrease in ATP concentration in cells exposed to fluorides [31,32,33,34,35].

In our previous study [36], we demonstrated that the application of NaF to THP1 macrophages at concentrations found in the blood serum of individuals exposed to environmental fluorides (1, 3, and 10 μM) led to a significant reduction in ATP synthesis. These findings suggest that fluoride may act as a respiratory chain uncoupler and affect cytochrome oxidase, as observed in our previous work [36]. It is possible that these effects are mediated through the inactivation of stress-activated kinases (Erk/MAPK) [37]. In the present work, we also noted a decreased amount of ATP in muscle CCL136 cells after NaF intoxication. The observed effect is also consistent with results obtained for other cell types: sperm cells [38], ertythrocytes [39,40], enamel cells [35], ovarian granulosa cells [41] or ameloblasts [42].

In many studies, the authors noted that muscle activation may be dependent on the muscle cell energy status, which is affected by glycogen supply and low glycogen level and is being linked with decreased muscle function [43,44,45,46]. Muscle contraction is a very complex process that is strongly dependent on the ATP level in the cell. The sequence of events begins by action potentials depolarizing the sarcolemma, which activates the voltage sensors in the triad junction and subsequently opens the channels in the sarcoplasmic reticulum to release Ca^2+^ to the cytoplasm [47]. The increase in the cytosolic concentration of Ca^2+^ from 10- to 20-fold leads to force generation in the muscle fibre. The ion pumps taking part in this mechanism: the Na/K-ATPase (sarcolemma) and the Ca-ATPase (sarcoplasmic reticulum) as well as the myosin ATPases at the contractile apparatus are directly dependent on ATP and are affected by low ATP or high Pi levels [30]. Moreover, Gejl et al. (2014) proved that muscle glycogen on the levels below approximately 300 mmol/kg dw may impair the rate of Ca^2+^ release from the sarcoplasmic reticulum vesicle and this level of glycogen is the minimum muscle glycogen content in untrained subjects in the human study [48]. Our study revealed that fluoride intoxication increased glycogen deposition in CCL136 cells. Changes in glycogen content in different cell and tissue types induced by fluoride are documented in several studies and the observed effects are ambiguous. Increased glycogen accumulation leading to atrophy was observed in chondrocytes in animals with fluorosis [49]. A similar effect was obtained for parathyroid gland cells in rats ingested with fluoride [50]. Fluoride was also shown to increase glycogen level in rabbit adrenal glands [16]. It was also shown that sodium fluoride increased muscle and liver glycogen in freshwater catfish, *Clarias batrachus* (Linn.) [17]. On the other hand, fluorine is also recognised as an inducer of glycogen degradation. Such activity was observed in hepatocytes [18,51,52], skeletal muscles [18] and in testicular tissue [53] in rats exposed to fluoride in the diet or by infusion. A decreased amount of glycogen was also observed in hepatocytes in rabbits with acute and chronic fluorosis [54], as well as in the spleen, lens, ovary, liver and skeletal muscle tissue of rabbits injected with sodium fluoride [55]. A decreased level of glycogen in gill, liver, kidney, and muscle tissue was also described for acute fluoride exposure of *Labeo rohita* fish [56]. No relationship between fluoride ingestion and hepatic glycogen level was concluded in the Zebrowski and Suttie study with fluoride-fed rats [57]. Interestingly, increased glycogen accumulation in salivary glands and liver was also found for lithium exposure [58].

Muscle glycogen synthase (GYS) is the rate-limiting UTP-requiring enzyme that catalyses the incorporation of UDP-glucose via α-1,4-glycosidic linkages into the growing glycogen polymer, with branching enzyme catalysing formation of α-1,6-branch points [9,59,60]. The activity of GYS is controlled by covalent modifications (phosphorylation by multiple kinases), allosteric activation by glucose-6-phosphate and compartmentation [59]. Insulin, together with exercise increases enzyme affinity for glucose-6-phosphate and for substrate [59,60]. GYS is inactivated by a complex, hierarchical multisite phosphorylation mechanism involving several protein kinases [61,62]. The observed decrease in glycogen content in CCL136 cells was accompanied, paradoxically, by lower gene expression and GYS protein content. However, this pattern of changes was statistically significant only at the highest concentration of NaF used. Basically, fluorides inhibit GYS activity [33,63]. Perhaps, however, the simultaneous lower expression of glycogen synthase kinases (GSK-3) compensates for the effect of lowering the expression and level of the GYS protein induced by fluoride.

GSK-3 (glycogen synthase kinase-3) plays a crucial role in regulating the phosphorylation of muscle GYS (glycogen synthase), which in turn affects its affinity for glucose-6-phosphate and UDP-glucose [59]. GSK-3 is a Ser/Thr protein kinase and is responsible for phosphorylating GYS. There are two isoforms of GSK-3, α and β, which share a high degree of similarity within their kinase domains but differ in their N- and C-terminal sequences. Both isoforms of GSK-3 (α and β) are known to have significant involvement in muscle metabolism [64]. Additionally, GSK-3 has been implicated in various other processes, including the regulation of protein synthesis, transcription of genes, and cell differentiation. Increased expression and activation of GSK-3 have been observed in the skeletal muscle of rodent models of obesity and obese individuals with type 2 diabetes, and this dysregulation is associated with impaired insulin-mediated glucose disposal and glycogen synthase activation [65]. Unlike most protein kinases, GSK-3 is typically active in unstimulated cells and is inhibited in response to a variety of inputs, including lithium, which is a relatively weak GSK-3 inhibitor [64]. GSK-3 catalytic kinase activity is controlled through differential phosphorylation of Ser/Thr residues (causing an inhibitory effect) or tyrosine residues (an activating effect). Negative phosphorylation of Ser 21 and Ser 9 is made through a phosphatidylinositol 3-kinase (PI3K)/protein kinase B (PKB/Akt)-dependent pathway. Additionally, GSK-3 is also associated with cAMP-dependent protein kinase A (PKA), which also phosphorylates Ser residues and inactivates both forms [66,67,68,69]. Fluoride is recognised as an inhibitor of many enzymes including kinases and phosphorylases [33,70]. Fluoride also appears to act as a regulating factor for muscle GSK, which was shown for the first time in our study. It seems that the observed decrease in both the non-phosphorylated and phosphorylated forms of this protein may result from the regulation of the gene expression. The altered pattern of gene expression is one of the molecular mechanisms of action of fluoride [71]. Fluorosis may be associated with increased gene expression and levels of PI3K and Akt1 proteins [72], the signaling of which regulates the state of GSK-3 phophorylation. Moreover, fluorine can induce the phosphorylation of Ser473 in Akt and Ser9 in GSK-3, resulting in inhibition of GSK-3 activity and consequently the maintenance of GYS in an active dephosphorylated form [73].

Glycogenolysis is regulated by glycogen phosphorylase (PYGM). This enzyme acts on the terminal α-1,4-glycosidic glucose residues, whereas the debranching enzyme targets the α-1,6-branchpoints in the glycogen molecule [9]. PYGM is regulated by phosphorylation of a single Ser residue at the N-terminus by phosphorylase kinase (PhK) in response to cAMP and calcium level signals. The activity of PYGM is also regulated by allosteric binding of a number of molecules [74]. The activity of PYGM is increased by allosteric binding of AMP and completed by ATP or glucose-6-phosphate, whereas Pi appears to limit PYGM activity at the substrate level [7]. Cyclic AMP is a second messenger used for intracellular signal transduction [75]. cAMP also regulates the passage of Ca^2+^ through ion channels [76]. In our previous work [13], we noted that in macrophages cultured with NaF, the concentration of cAMP significantly increased in a dose-dependent manner even 221% for 10 μM NaF. This is probably due to the influence of fluoride, which increases cAMP production by stimulating adenylate cyclase [76], where the enzyme itself catalyses the production of cAMP from ATP [77]. Moreover, water fluoride is positively correlated with serum cAMP concentration—it was shown that fluoride has a positive, dose-dependent effect on serum cAMP concentration in patients with fluorosis [78]. In such context, fluoride should have a stimulatory effect on PYGM activity [33,79,80]. On the molecular level, reduced expression, and thus, lower content of PYGM protein forms may respond to increased glycogen deposition observed in the CCL136 cells. Acute fluoride toxicity in the rat is accompanied by a marked hyperglycemia, the magnitude of which is dose-dependent [18]. The authors noted that, besides muscle and liver glycogen contribute to the increased blood glucose, there was no direct effect of fluoride on liver glycogen phosphorylase [18].

In summary, NaF intoxication may lead to multi-level disturbances in muscle cells. One of the most serious effects is glucose storage metabolism disruption. Fluorine is known to modulate the activity of glucose and glycogen-related enzymes, but so far little is known and the data are not complex. To the best of our knowledge, the present study is the first of its kind to describe the complex effects of low fluoride levels on the gene expression of enzymes involved in glycogen metabolism.

## 4. Materials and Methods

### 4.1. Cell Culture Preparation

Cells from the CCL136 muscle cell line (LGC Standards, Łomianki, Poland) were seeded in 6-well plates at a density of 2 × 10^6^ cells/well. The cells were cultured for 48 h at 37 °C in a humidified atmosphere of 95% air and 5% CO_2_ in Dulbecco’s Modified Eagle’s high glucose Medium (DMEM; Sigma-Aldrich, Poznań, Poland) supplemented with 10% fetal bovine serum (FBS; Gibco, Billings, MT, USA) and antibiotics (100 U/mL penicillin and 100 mg/mL streptomycin; Sigma-Aldrich, Poznań, Poland). The DMEM contained 4500 mg/L of glucose. NaF (sodium fluoride) was added to the culture medium at final concentrations of 1, 3 and 10 µM. The selection of NaF concentrations was based on previous studies that determined fluoride levels in human serum (0.75–1.75 µM for a healthy person and 6.5 µM for a person with fluorosis) [81,82]. After the incubation period, insulin was added to the cells at a concentration of 0.1 µM for 30 min. The cells were then harvested by scraping and centrifuged at 250× *g* for 5 min to obtain a pellet. Cell viability was assessed using the trypan blue dye exclusion method, and the cell count was determined using a Bright Line Hemacytometer (Sigma-Aldrich, Poznań, Poland). Only cell cultures with a viability of more than 97% were used for the experiments. Protein concentration was measured using the Micro BCA Protein Assay Kit (Thermo Scientific, Waltham, MA, USA).

### 4.2. Fluorimetric Assay: Intracellular Glycogen Accumulation—Quantitative Analysis

After incubation with NaF and insulin, the cells were scraped from the plate and immediately centrifuged (250× *g*/5 min/4 °C). Next, Assay Buffer containing protease inhibitors was added to the cell suspensions, and the samples were frozen (−20 °C) for 10 min. Subsequently, the samples were centrifuged (800× *g*/10 min/4 °C), and the supernatants were collected for analysis. The determination of intracellular glycogen accumulation was performed using the Glycogen Assay Kit (Cayman Chemicals, New York, NY, USA) following the manufacturer’s protocol. In this assay, amyloglucosidase hydrolyzes glycogen to form β-D-glucose, which is then specifically oxidized by glucose oxidase to D-glucono-δ-lactone, resulting in the production of hydrogen peroxide. Hydrogen peroxide, in the presence of horseradish peroxidase, reacts with 10-acetyl-3,7-dihydroxyphenoxazine (ADHP) in a 1:1 stoichiometry, generating the highly fluorescent product resorufin. The fluorescence of resorufin is measured at an emission wavelength of 585–595 nm.

### 4.3. Fluorescent Microscopy: Imaging of Glycogen Accumulation Using PAS (Periodic Acid Schiff) Method

Cells from the CCL136 cell line, in a quantity of 5 × 10^5^, were incubated on microscope slides with various fluoride solutions according to the previously mentioned procedure. The incubation took place in a humidified atmosphere of 95% air and 5% CO_2_ at 37 °C for 48 h. Following the cultivation period, the cells were rinsed three times with PBS and then incubated for 5 min with a mixture containing periodic acid, 96% ethanol, 0.5 M sodium acetate, and deionized water. Subsequently, the cells were washed with 70% ethanol and incubated for 30 min with Shiff’s reagent at room temperature in the dark. Afterward, the cells were rinsed with a reduction reagent, which consisted of a mixture of KJ and sodium thiosulfate with the addition of 1 N HCl and 96% ethanol. Finally, the cells were washed with deionized water, and the cell nuclei were differentiated using acidic hematoxylin. The prepared slides were examined under a fluorescence microscope (Axioskop, Carl Zeiss, Oberkochen, Germany) with the aid of filter 09 (Filter set 09–487909–0000; Carl Zeiss, Oberkochen, Germany) following the protocol described by Tabatabaei Shafiei et al. in 2014 [83].

### 4.4. High Performance Liquid Chromatography: Intracellular ATP Level—Quantitative Analysis

During the 48-h incubation period with NaF solutions, the HPLC separations were conducted using an Agilent Technologies 1260 liquid chromatograph system (Warsaw, Poland). The system consisted of a G1379B degasser, a G1312B binary pump, a G1316A column oven and a G1315C DAD VL+ detector. Samples were injected using a G1329B autosampler. Instrument control, data acquisition and analysis were performed using Agilent ChemStation software (version offline) (Agilent Technologies, Cheadle, UK). The separation was carried out on a Thermo Scientific Hypersil BDS C18 column with dimensions of 100 × 4.6 mm and a particle size of 3 µm. The column oven temperature was set to 20 °C. To achieve appropriate separation of all analytes of interest, a dual mobile phase gradient was employed. Mobile phase A consisted of 150 mM KH_2_PO_4_/K_2_HPO_4_ and 150 mM KCl at pH 6.0. Mobile phase B had the same final concentrations as mobile phase A, with the addition of 15% acetonitrile (*v*/*v*). The flow rate was maintained at 1.0 mL/min, and the injection volume for each sample was 20 µL. Peaks were monitored by the DAD detector at an adsorption wavelength of 254 nm.

### 4.5. Real-Time PCR Analysis—Glycogen-Related Enzymes Genes Expression Level

#### 4.5.1. RNA Extraction and cDNA Synthesis

The extraction of RNA was conducted using the RNeasy Mini Kit from Qiagen (Wrocław, Poland), following the manufacturer’s instructions. Subsequently, cDNA synthesis was performed using the SuperscriptTM II RT kit from Invitrogen (Legnica, Poland), also following the manufacturer’s instructions.

#### 4.5.2. Quantification of Gene Expression Using Real-Time PCR

For quantitative real-time PCR, a 7500 Fast Real-Time PCR System from Applied Biosystems (Warsaw, Poland) was utilized. FAM-labeled probes specific to glycogen synthase (GYS-1; Hs00157863), GSK3A (Hs00997938), glycogen phosphorylase (PYGM; Hs00989942) and the control gene CYCLO (Hs99999904) were obtained from Applied Biosystems. PCR reactions were performed in duplicate with a total volume of 20 µL, consisting of 10 µL of TaqMan^®^ Gene Expression PCR Master Mix, 2 µL of cDNA and 1 µL of the respective assay. The fluorescence data obtained were analysed using 7500 Software v2.0.2 (Applied Biosystems).

The relative expression of the target gene was calculated using the ΔΔCt method of relative quantification. The fluorescence data were analysed using 7500 Software v2.0.2. The ΔΔCt method was employed to compare the relative expression of the target gene under experimental conditions to that of the control conditions, as described in the Applied Biosystems manual.

### 4.6. Western Blotting Analysis—Glycogen-Related Enzymes Protein Level

Following incubation with NaF, the cells were washed with phosphate-buffered saline (PBS) from PAP Laboratories (Warsaw, Poland) and lysed using a lysing buffer containing protease inhibitors, 1% sodium dichloroisocyanurate, 5 mM ethylene-diaminetetra-acetic acid, 1% Triton-X 100, 100 mM sodium orthovanadate from Sigma-Aldrich (Poznań, Poland), and PhosStop from Sigma-Aldrich (Poznań, Poland). The protein concentration in the obtained cell lysates was determined using the Micro BCA Protein Assay Kit from Thermo Scientific (Pierce Biotechnology, Waltham, MA, USA). Equal amounts of protein were separated on a 10% sodium dodecyl sulfate (SDS)/polyacrylamide gel using electrophoresis and subsequently transferred to a nitrocellulose membrane from Thermo Scientific (Pierce Biotechnology, Waltham, MA, USA) at a constant current of 157 mA for 1.5 h at room temperature (RT). The membrane was then blocked with 5% non-fat milk (for GSK-3α/β, pGSK-3α, PYGM) or 3% non-fat milk (for GYS-1, pPYGM) in Tris-buffered saline from Sigma-Aldrich (Poznań, Poland) containing 0.1% Tween 20 from Sigma-Aldrich (Poznań, Poland) for 1 h at RT. Subsequently, the membrane was incubated with primary antibodies against GYS-1 (1:200; Santa Cruz Biotechnology, Santa Cruz, CA, USA), GSK-3α/β (1:200; Santa Cruz Biotechnology, Santa Cruz, CA, USA), pGSK-3α (1:200; Santa Cruz Biotechnology, Santa Cruz, CA, USA), pGSK3A (1:200; Santa Cruz Biotechnology, Santa Cruz, CA, USA), PYGM (1:200; Santa Cruz Biotechnology, Santa Cruz, CA, USA), pPYGM (1:200; Santa Cruz Biotechnology, Santa Cruz, CA, USA) or with a monoclonal anti-ß-actin antibody (1:200, clone AC-74, Sigma-Aldrich). Following primary antibody incubation, the membrane was incubated with secondary antibodies (1:5000; goat anti-rabbit for GYS-1, PYGM, pPYGM, and goat anti-mouse for GSK-3, pGSK-3 and ß-actin). The signals were visualised using chemiluminescence detection from Thermo Scientific (Pierce Biotechnology, Waltham, MA, USA).

The bands corresponding to the individual samples and the control protein (beta actin) were analysed quantitatively using image analysis software. The levels of the control protein in each loading lane were used to normalize the target protein levels analysed in each assay. Normalization of the intensity of the bands of the analysed protein consisted in dividing their values by the appropriate values of the intensity of the bands of the protein that controls the loading. This normalization method corrects for any differences in protein loading or transfer efficiency between different samples.

By normalizing target protein levels to loading control protein levels, data analysis takes into account any discrepancies that may have occurred during the experimental process. The normalized data were then subjected to statistical analysis and presented in a graphical form.

### 4.7. Immunohistochemistry

Muscle cells cultured on cover slides from Thermo Scientific (Menzel Glaser, Braunschweig, Germany) were fixed using 4% buffered formalin. To expose the epitopes, the fixed cells were treated with 0.1% Triton X-100 from Merck (Warsaw, Poland) for 20 min. After washing with phosphate-buffered saline (PBS), the endogenous peroxidase activity was blocked using a 3% solution of perhydrol in methanol. Subsequently, the cells were incubated at room temperature (RT) for 60 min with the following primary antibodies: polyclonal antibody against GSK-3α/β (Santa Cruz Biotechnology, Santa Cruz, CA, USA; final dilution 1:500) and pGSK-3α (Santa Cruz Biotechnology, Santa Cruz, CA, USA; final dilution 1:500), mouse monoclonal antibody against PYGM (Abcam, Cambridge, UK, final dilution 1:500), and antibody against GYS-1 (Santa Cruz Biotechnology, Santa Cruz, CA, USA; final dilution 1:500). To visualise the antigen-antibody complex, the Dako LSAB+System-HRP (DakoCytomation, Glostrup, Denmark, Code K0679) was used, which involves the reaction of avidin-biotin-horseradish peroxidase with DAB as a chromogen, following the staining procedure instruction provided. The slides with the treated cells were gently washed with distilled water and counterstained with hematoxylin. The cover slips containing the stained cells were mounted on histological microscope glass slides from Thermo Scientific (Menzel Glaser, Braunschweig, Germany). For the negative control, specimens were processed without the addition of a primary antibody. Positive staining was identified microscopically (Leica DM5000B, Wetzlar, Germany) by visual observation of brown pigmentation.

### 4.8. Determination of Protein Concentration

Protein concentration in the cells was determined using the Micro BCA Protein Assay Kit from Thermo Scientific (Pierce Biotechnology, Waltham, MA, USA) and a spectrophotometer (UVM340, ASYS, Biogenet, Józefów, Poland). The bicinchoninic acid (BCA) protein assay was performed following the instructions provided by the manufacturer. This assay kit is composed of two components, a high-precision, detergent-compatible assay reagent set, designed to measure the total protein concentration relative to a protein standard. The assay method combines the reduction of Cu^2+^ to Cu^1+^ by proteins in an alkaline medium with the colorimetric detection of the cuprous cation (Cu^1+^) using bicinchoninic acid, which offers high sensitivity and selectivity.

### 4.9. Statistical Analysis

Statistical analysis of the obtained results was conducted using the Statistica 12 software package from Stat Soft (Kraków, Poland). The arithmetical mean and standard deviation (SD) were calculated for each of the parameters under study. Since the distribution of variables deviated from normal distribution in most cases, as determined by the Shapiro-Wilk test, non-parametric tests were employed for further analysis. For related samples, the significance was initially assessed using Friedman’s analysis of variance, and if significant results were obtained, the Wilcoxon matched-pair test was applied. Statistical analysis of gene expression was performed using the Fisher test. All tests were evaluated at a significance level of *p* < 0.05. 

## Data Availability

Available at request.

## Abbreviations

ATP	adenosine triphosphate
BCA	bicinchoninic acid
Ca-ATPase	calcium dependent transmembrane ATPase
CCL136	muscle cell line
DMEM	Dulbecco’s Modified Eagle’s high glucose Medium
FBS	fetal bovine serum
GSK-3α/ß	glycogen synthase kinase 3
GSK-3α/β	glycogen synthase kinase,
GYS	glycogen synthase
GYS-1	muscle isoform of glycogen synthase,
HPLC	High Performance Liquid Chromatography
Na/K-ATPase	sodium/potasium pump, an electrogenic transmembrane ATPase
NaF	sodium fluoride
PBS	phosphate-buffered saline
Pgsk	3α–glycogen synthase kinase, phosphorylated form
PhK	phosphorylase kinase
PI3K	phosphatidylinositol 3-kinase
PKA	cAMP-dependent protein kinase A
PKB/Akt	protein kinase B
pPYGM	muscle isoform of glycogen phosphorylase, phosphorylated form
PYGM	glycogen phosphorylase
PYGM	muscle isoform of glycogen phosphorylase
ROS	reactive oxygen species
